# Barriers and Facilitators of Help-Seeking for LGBTQ+ Survivors of Sexual Violence: A Systematic Review

**DOI:** 10.1177/15248380251355902

**Published:** 2025-08-17

**Authors:** Ciara Buckley, Aiswarya Radhakrishnan, Lorraine Boran, Maggie Brennan, Áine Travers

**Affiliations:** 1 Dublin City University, Ireland

**Keywords:** LGBTQIA+, sexual violence, victims/survivors, help seeking/reporting/disclosure, race/ethnicity

## Abstract

People who identify as LGBTQ+ (Lesbian, Gay, Bisexual, Transgender, Queer, plus) are known to experience similar or higher levels of sexual violence compared to their heterosexual cisgender counterparts. However, sexual violence research has largely focused on heterosexual female survivors of male perpetrated crime. Thus, the unique support needs and help-seeking patterns of LGBTQ+ survivors are poorly understood. This review addresses this gap by systematically exploring literature on barriers and facilitators to help-seeking for LGBTQ+ survivors of sexual violence. Four databases (PsycINFO, CINAHL, MEDLINE, and Web of Science) were searched to identify relevant material, with 35 articles (30 qualitative, 1 quantitative, and 4 mixed-methods) meeting the inclusion criteria. Data were extracted and analyzed using a narrative synthesis. The topic was investigated almost exclusively cross-sectionally. Barriers included discrimination experiences, myths and stereotypes, feelings of shame and self-blame, and rejection of victim status. Additional barriers were reported by survivors who hold multiple minority identities, in particular LGBTQ+ people of color and sex workers. Facilitators to help-seeking included the intrinsic need to connect with others, social encouragement and empowerment, and positive disclosure experiences. The Power Threat Meaning framework provides insight into these findings by presenting help-seeking behaviors as adaptive responses to increase a sense of safety following a traumatic experience. The analyzed data indicate several implications for the development and improvement services to support LGBTQ+ survivors. They further serve to highlight the need for additional robust research, conducted with an intersectional lens, to explore the needs of sexual and gender minority survivors of sexual violence.

## Introduction

Sexual violence is a pervasive global problem and is recognized by the World Health Organization (WHO) as a major threat to health and well-being (WHO, 2021). Exact global prevalence rates are difficult to ascertain due to problems such as widespread under-reporting and differences in data collection practices across countries (WHO, 2021), as well as a tendency of large epidemiological surveys to sample women only (Walby et al., 2017). U.S. estimates suggest that more than half of women and almost a third of men experience sexual violence in their lifetime, with a disproportionately higher prevalence among minority groups (Basile et al., 2022). The term “sexual violence” includes crimes like rape, sexual abuse, image-based abuse, harassment, exploitation, or coercion (WHO, 2021). Risk seems to vary based on sexuality and gender; for example, epidemiological survey data from the United States (*n* = 41,174; Chen et al., 2020) found bisexual women and lesbians experience significantly higher rates of sexual violence than heterosexual women. Similarly, Hoxmeier (2016) found that transgender individuals were significantly more likely to experience victimization than their cisgender counterparts (*n* = 19,639). Researchers (e.g., Anderson et al., 2021) have identified a need to develop the evidence-base relating to LGBTQ+ experiences through the lens of minority stress theory (Meyer, 1995, 2003, Meyer & Frost, 2013).

Sexual violence is often a highly traumatic experience with long-term negative implications for survivors including post-traumatic stress disorder (PTSD), anxiety, depression, and psychological distress (Geppert et al., 2024). This can result in survivors finding it difficult to maintain relationships (Stockman et al., 2024) or employment (Loya, 2015). To recover and heal from experiences of sexual violence, survivors can seek help from formal services (e.g., police or legal advocacy, rape crisis centers, medical personnel, or counselors) and informal sources (e.g., friends, family, and romantic partners; Dworkin et al., 2019). Despite the high prevalence of sexual violence in the LGBTQ+ community, research into victimization and help-seeking typically focuses on heterosexual cisgender populations, and comparatively little is known about LGBTQ+ people’s experiences (Ollen et al., 2017). Moschella et al. (2020) found that bisexual survivors are significantly more likely to disclose victimization and report significantly lower well-being, and life satisfaction post-victimization in comparison with lesbian, gay, or heterosexual people. In evaluating recovery outcomes of women, Sigurvinsdottir and Ullman (2015) reported greater PTSD symptomology in lesbian and bisexual women compared with heterosexual participants. In addition, bisexual women demonstrated elevated depression symptoms and exhibited more problematic substance use. In comparing women’s experiences by orientation, Long et al. (2007) found that bisexual women disclosed victimization to significantly more formal sources (71.4%) compared to lesbian (63.3%) or heterosexual participants (58.2%) yet received the fewest positive disclosure responses and exhibited significantly more symptoms of depression and PTSD.

Research on factors influencing this phenomenon is in its infancy; however, existing theoretical frameworks can offer insight into the area. Minority stress, as outlined by Meyer (1995, 2003), Meyer and Frost (2013), suggests that LGBTQ+ people experience stressors related to discrimination and prejudice linked with homophobia and cisnormativity. Moschella et al. (2020) proposed that minority stress may compound the trauma of sexual victimization for lesbian, gay, and bisexual survivors of sexual violence; therefore, LGBTQ+ people may be more likely to seek support than heterosexual survivors because it may push them to a tipping point in the context of existing stressors. Minority stress may also explain lower levels of satisfaction with responses to disclosure as noted by Long et al. (2007). Karakurt et al. (2024) found a lack of LGBTQ+ representation makes navigating gendered services particularly difficult for LGBTQ+ survivors.

LGBTQ+ survivors who are members of ethnic and racial minority groups may experience increased levels of minority stress, as well as experiencing minority stress in specific ways, due to their intersecting marginalized identities (Cyrus, 2017). Intersectionality is a framework for analyzing social power relations and imbalances, relating to how different aspects of an individual’s identity can intersect to produce specific experiences of power and marginalization in different contexts (Bowleg, 2008; Bowleg et al., 2023; Combahee River Collective, 1977; Crenshaw, 1989, 2005). Racially discriminatory power dynamics affecting people of color have transcended generations and are built upon on a history of de-valuation, resulting in the de-prioritization of ethnic minority groups (Bowleg et al., 2023; Combahee River Collective, 1977). Crenshaw (1989) asserts that this devaluation influenced the very formation of U.S. sexual violence laws, as they were rooted in protecting female chastity, a concept reserved for white women, excluding Black women from a need for protection. They also categorized sexual violence as a property crime (Martin et al., 2007), perpetrated by one man against another, thereby neglecting male victimization.

The frameworks of minority stress and intersectionality provide a vocabulary to describe and understand the ways in which people with marginalized identities receive messages of discrimination and disempowerment throughout their daily lives, and how these messages can be internalized. As described in the Power Threat Meaning Framework (PTMF; Johnstone & Boyle, 2018), the way a person perceives, makes meaning of and responds to a traumatic event is shaped by their social world and how they see themselves within it. It suggests that individuals evolve within their cultural context, adapting in a way that optimizes survival. The PTMF moves away from pathologizing those who experience adversity, instead treating their behavior and emotions as rational responses to negative life events. Those disadvantaged by power imbalances will, therefore, likely exhibit distinct responses through their adaptation to a system that marginalizes and discriminates against them. This theory may serve to explain why the limited data available suggests that the consequences of sexual violence survivorship may be more severe for sexual minority individuals compared to their heterosexual counterparts. The PTMF has been recently utilized by Brett et al. (2024) to explain the observation that LGBTQ+ individuals faced with cisheteronormative systems may feel pressured into self-censorship and concealment of their marginalized identity. Sexual violence support networks can also reflect these exclusionary messages. Guckenheimer (2021) highlights that, despite many Rape Crisis Centres’ efforts to include transgender survivors, their historical exclusion continues to reinforce gender inequality experienced by this community.

Sexual violence support workers are highly likely to encounter LGBTQ+ service users; however, the scarcity of data relating to this population means it may be difficult for some providers to understand and meet their needs. As such, this review aims to explore the literature and provide a synthesis of barriers and facilitators to help-seeking for LGBTQ+ survivors of sexual violence. It is intended that this can be utilized to enable policymakers, practitioners, and researchers to better understand and meet the needs of these users.

## Methodology

This study was conducted in line with best practice for systematic reviews as outlined in the Preferred Reporting Items for Systematic Reviews and Meta-Analyses (PRISMA) framework. The PRISMA checklist (Page et al., 2021) guided the design of the review, and the presentation of results. This study was registered with Prospero (ID CRD42024509382).

### Study Selection

**Figure 1.**
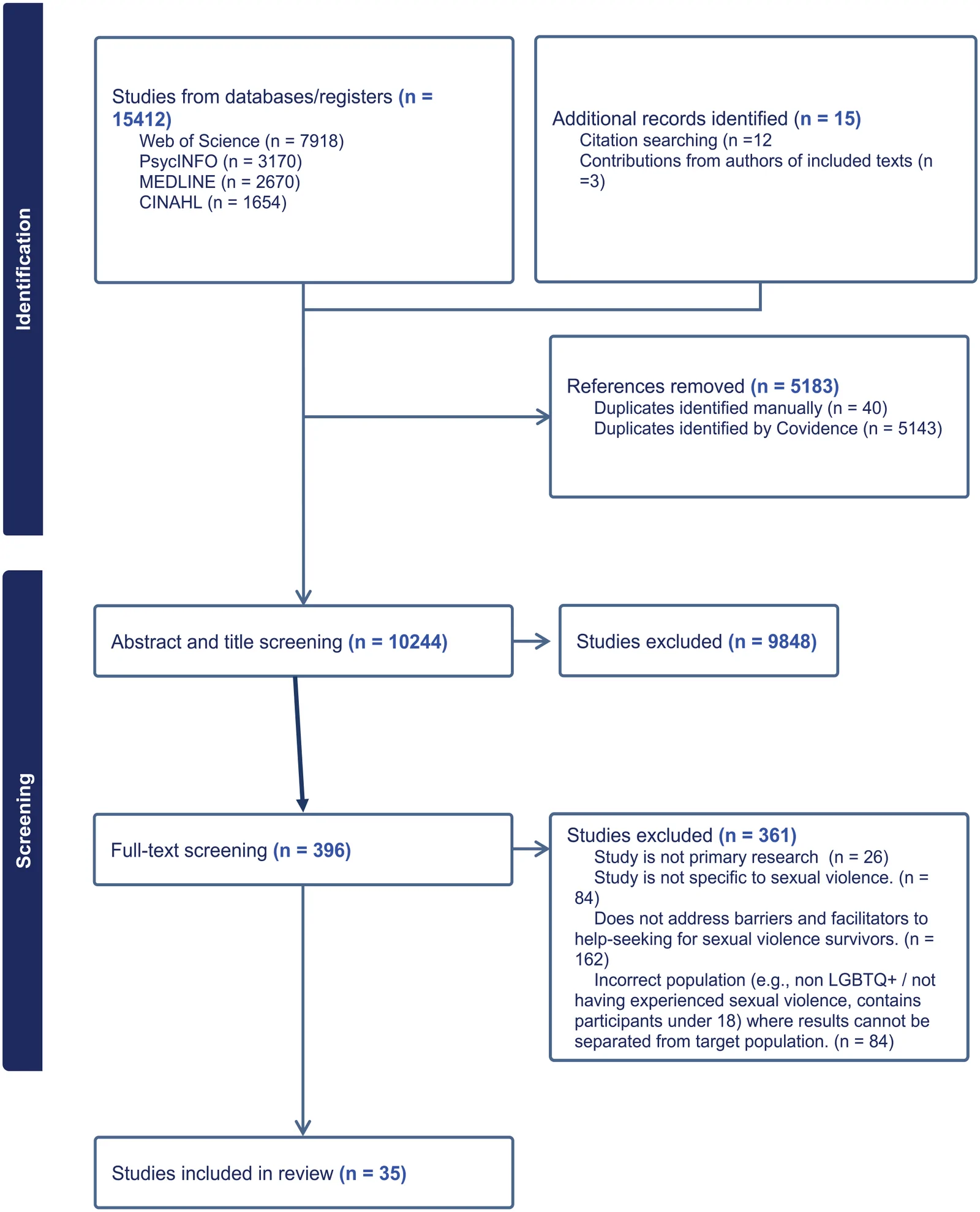
PRISMA flow diagram. *Note*. PRISMA = Preferred Reporting Items for Systematic Reviews and Meta-Analyses.

Searches were carried out on February 15, 2024, across four databases (PsycINFO, CINAHL, MEDLINE, and Web of Science). No time restrictions were applied to the search. Three broad categories of search terms were established: (a) LGBTQ+, (b) survivors of sexual violence, and (c) help-seeking. The full search strategy is provided in the Supplemental File. The initial search returned 15,412 articles, which were uploaded to the systematic review screening platform Covidence. An additional 15 articles were identified through citation searching and inviting contributions from authors of included texts. Removal of duplicates resulted in 10,244 articles remaining for title and abstract screening. The first and second authors independently screened each abstract. Eligibility criteria required studies to be (a) primary research, (b) specific to LGBTQ+ survivors of sexual violence, (c) address barriers and facilitators to help-seeking, and (d) involve participants over the age of 18. Articles were removed if they were not written in English. Following title and abstract screening, 396 articles remained for full-text screening. This was also carried out independently by the first and second author, with conflicts in ratings resolved through discussion. Articles were removed if they did not offer insight into LGBTQ+ sexual violence survivors’ reasons for engaging or abstaining from support following an experience of sexual violence. For example, in some cases, articles were excluded as only support workers’ perspectives were included. In total, 35 articles met the inclusion criteria for analysis. See Figure 1 for full detail of the screening process.

### Quality Appraisal

Quality appraisal was carried out prior to data extraction to minimize the potential of bias. Each study design was assessed by an appropriate tool, namely the Critical Appraisal Skills Programme (CASP) for qualitative studies, the Quality Review and Critical Appraisal method (Dunne et al., 2017) for quantitative studies, and the Mixed Methods Appraisal Tool (MMAT) developed by Hong et al. (2018) for mixed methods studies.

### Data Extraction

Data were extracted into a prespecified extraction table in line with the PRISMA methodology (Liberati et al., 2009) 27-item checklist and flow design. The table incorporated the headings: Source, location, design, quality assessment, aim, analysis, LGBTQ+ subgroup included, participant details, main findings, barriers, and facilitators (summarized in Table 1). In addition, a narrative synthesis (Mays et al., 2005) is presented below.

**Table 1.** Study Characteristics and Main Results.

Source	Sample Details	Main Findings	Barriers Identified	Facilitators Identified
Study 1. Mennicke et al. (2025). United States. Qualitative. Open survey.	College survivors (*N* = 273, *M* age 23). Woman (*n* = 157), Man (*n* = 100), transgender (*n* = 5), other (*n* = 11). Orientation: gay (*n* = 17), Bisexual (*n* = 44), straight (*n* = 168), other (*n* = 26).	LGBTQ+ students face many barriers to accessing support including, dismissal by service providers, lack of knowledge, poor reputation.	Embarrassment Fear their experience will be minimized Uncertainty about reporting procedures Discouragement from service providers	
Study 2. Walsh et al. (2024). United States. Qualitative. Interviews	Students (*N* = 29, age 18–35) who were LGBTQ+, racial minorities or both. Man (*n* = 3), woman (*n* = 22), transgender, non-binary, questioning (*n* = 4). Heterosexual (*n* = 12), queer (*n* = 10), questioning lesbian, bisexual, pansexual, (*n* = 7)	LGBTQ+ people and people of color (POC) face difficulty labeling an incident, limited access to affirming care and lack of knowledge about resources.	Uncertainty about reporting procedures Lack of peer-led services long wait lists Not wanting to navigate services alone Guilt about taking a space with a service	Being a member of an affinity group (i.e., group for ethnic minority students) where they felt connected and relaxed.
Study 3. Tan et al. (2021). Singapore. Qualitative. Interviews	Singapore residents (*N* = 33, age 21–50) with a history of sexualized drug use	Sexualized drug use can be a coping mechanism and can facilitate emotional connection	Fear of being reprimanded for sexualized drug use.	
Study 4. Guadalupe-Diaz and Jasinski (2017). United States. Qualitative. Interviews	Transgender IPV survivors (*N* = 18, age 18–49). Participants self-described themselves with each identity being within the transgender subgroup	The cisgender response to sexual violence survivors can be a barrier for transgender survivors and may complicate accepting victimization	Police misconduct/fear of prosecution Not feeling like a priority or “real” victim Feeling of having no one to tell Fear of being invalidated or discriminated Difficulty integrating victimization and masculinity	
Study 5. Carlisle & Schmitz (2023). United States. Qualitative. Interviews.	Adult Male college sexual violence survivors (*N* = 29). Orientation: gay or reported sex with men, heterosexual, asexual (*n* = 1). Transgender (*n* = 1).	Male sexual violence survivors may struggle to process sexual violence experiences and to describe incidents.	Difficulty linguistically processing sexual violence and labeling an incident as rape/sexual violence Fear recipient will normalize the experience Fear of victim-blaming	
Study 6. Hereth (2021). United States. Qualitative. Interviews.	Participants (*N* = 21) were transgender and assigned male at birth (age 20–33). Described as “transgender woman,” “woman” and “female,” or genderqueer.	Narratives reveal persistent traumatic experience over the lifetime, including child abuse, IPV and discrimination.	Belief that their case will not result in prosecution Fear of not being believed Fear of persecution due to sex work Belief in perpetrator regret	Social encouragement Engaging with other survivors Feeling cared for and connected to others
Study 7. Strauss Swanson and Szymanski (2020). United States. Qualitative. Interviews.	Sexual violence survivors and activists (*N* = 16, age 20–73). Gender: Female, genderqueer/gender non-conforming or male. Orientation: bisexual, gay/queer, queer and .heterosexual	Activism helped participants regain their power following victimization, helping others can be a way to heal and mainstream movements can increase awareness.	Feelings of shame Feelings of self-blame Feeling they do not meet societal expectations of a survivor (e.g., drinking, knowing the perpetrator).	Evolving understanding Desire to channel rage To connect with others Building comfort slowly To make societal difference
Study 8. Fernández-Rouco et al. (2017). Spain. Qualitative. Interviews.	Transgender survivors (*N* = 33, age 18–52). Transgender men (*n* = 8) or transgender woman (*n* = 25).	High levels of sexual violence were present as well as reliance on avoidance coping mechanisms.	Fear of not being believed Embarrassment Not wanting to share their transition details	Desire to obtain protection from the perpetrator
Study 9. D. Meyer (2020). United States. Qualitative. Interviews.	LGBTQ+ men (*N* = 21, age 22–77) who reported sexual violence to the police. Orientation gay (*n* = 15), bisexual (*n* = 4), queer (*n* = 2).	Fear of police discrimination was associated with non-feminine presenting and older participants.	Past experiences of being racially profiled by police resulting in distrust and expectations of discrimination.	Practical reasons; to obtain access to their home seeking protection
Study 10. Sherman et al. (2022). United States. Qualitative. Interviews.	Black or multiracial (including Black) transgender adult women (*N* = 19). who were assigned male at birth	Coping strategies were similar to cis participants but with increased self-affirming behavior.	Feeling of being victim blamed by police, leading to disengagement and a refusal to pursue a case.	To gain legal protection To ensure safety Desire to prosecute the perpetrator
Study 11. Brandt and Stults (2023). United States. Qualitative. Interviews.	Sexual minority men (*N* = 25, age 23–28) and had had sex with a male partner. Orientation: gay (*n* = 20), bisexual (*n* = 4) and queer (*n* = 1).	Coping strategies include help-seeking, distraction, cognitive reappraisal, venting, denial and avoiding future relationships.	Not wanting to manage feelings of others Fear recipient will minimize experiences Unsupportive/blaming responses from friends The belief they won’t be understood	Automatic thinking following the incident Wanting to be around others and connect
Study 12. Asseervatham et al. (2023) Cambodia. Qualitative interviews.	Transgender Cambodian Women (*N* = 20, age 21–49) who had experienced gender-based violence.	Barriers to formal supports include stigma consciousness, internalized stigma, low self-esteem, lack of knowledge, cost and lack of support.	Internalized stigma and self-blame Financial difficulties Lack of knowledge about services and reporting Believing services cannot help sex workers	Advice from friends
Study 13. G. D. Anderson and Overby. (2020) United States. Qualitative. Interviews.	Sexual violence survivors and service providers (*N* = 19, age 21–75). Orientation: female (88%), transgender or nonbinary (12%).	Support workers are not immune to barriers faced by the wider community and face additional challenges inhibiting engagement.	Fight-or-flight response of denial to self-protect and maintain current levels of functioning	
Study 14. Neves et al. (2023). Portugal Qualitative. Interviews	LGBTQ IPV survivors (*N* = 16, *M* age 31.4). Male (*n* = 5), Female (*n* = 9), non-binary (*n* = 2). Lesbian (*n* = 4), gay (*n* = 4), pansexual (*n* = 3), bisexual (*n* = 2), straight (*n* = 2).	Sexual violence was highly prevalent, transgender participants reported more instances: coercion, threats manipulation and deprivation	Believing childhood sexual activity to be normal Believing the perpetrator would not remember the incident	
Study 15. Turner and Hammersjö 2024). Sweden. Qualitative. Interviews.	IPV survivors (*N* = 7, age 18–58) who have or have wanted to engage support. Gender: transgender male, woman, queer, non-binary. Orientation: homosexual, queer, bisexual.	LGBTQ+ IPV survivors face doubts and worries about seeking support and navigating support.		Effective service providers (i.e., who normalize the experiences and validate them in a non-judgmental way)
Study 16. Mgolozeli and Duma (2020) South Africa Qualitative. Interviews.	Sexual violence survivors who reported to police (*N* = 21, age 18–65). Orientation: homosexual (*n* = 3) or heterosexual (*n* = 8).	Most men were motivated to report rape to the police despite barriers and negative past experiences.	Negative experiences with police Fear of retribution from the perpetrator Fear of victim-blaming and mockery Thinking male rape will not be believed Uncertainty around reporting requirements	To obtain protection To understand why they were victimized Social encouragement Anger at the perpetrator
Study 17. de Heer et al. (2023). United States. Qualitative. Interviews.	LGBTQ+ survivors (*N* = 15): lesbian woman (*n* = 5), queer woman (*n* = 4), bisexual woman (*n* = 2), queer transgender (*n* = 1), queer gender non-conforming (*n* = 2), queer man (*n* = 1)	LGBTQ+ sexual violence survivors who experience negative reactions to disclosure may refrain from engaging in help-seeking.	Unsupportive disclosure responses (blame) Advice from police/lack of faith in police Embarrassment of LGBTQ+ stereotypes Fear of ignorance of LGBTQ+ issues Difficulty categorizing their experience Heteronormative understanding of rape	Competent support workers (therapist experienced in trauma & LGBTQ+ concepts who can validate both the experience and identity).
Study 18. Bedera et al. (2023). United States. Qualitative. Interviews.	Queer women (*N* = 40, age 18–31) who identified as she/her and genderqueer (*n* = 1). Queer (*n* = 12), bisexual (*n* = 18), lesbian (*n* = 6), pansexual (*n* = 3), demisexual (*n* = 1)	Reactions to disclosure can be detrimental to well-being and help-seeking, with more significant impact for queer, survivors.	Reluctance to tarnish LGBTQ+ culture Fear of being blamed or minimization Fear disclosure will delegitimize sexuality Fear parents will hinder help-seeking Discouragement from family members	Having people who supported them previously (with their sexual identity)
Study 19. Coston. (2019). United States. Qualitative. Focus groups.	LGBTQ+ sexual violence survivors (*N* = 38). Transgender woman, non-binary trans-masculine individual, queer black man, queer black trans woman and a genderqueer person.	Queer and trans people of color (QTPOC) exhibit low levels of awareness of sexual violence which inhibits engagement with supports. Survivors fear discrimination based on race, orientation and gender.	Past experiences of homophobia or racism Concerns for the perpetrator or fear of retribution Experiencing ridicule from providers Inability to categorize an experience Confidentiality concerns/internal shame	Feeling empowered Competent support worker Having a safe space Being allowed to set the pace Desire to learn more
Study 20. Hawkey et al. (2021). Australia. Qualitative. Interviews and photovoice submission.	Transgender women of color (*N* = 31, age 18–54). Orientation included: straight, pansexual, bisexual, *N*/A, queer, lesbian, asexual, curious, panromantic/demisexual, pansexual leaning toward women.	Sexual violence is associated with fear, anxiety and depression. Survivors restrained their movements and were hypervigilant to prevent re-victimization.	Lack of similar people in support networks (people of color [﻿POC]) and LGBTQ+ groups Being denied support when asking for it Experiencing victim blaming Exclusion from women’s support groups Seeing formal support as inaccessible or inadequate	To alleviate trauma symptoms To demonstrate resilience Community support Mainstream visibility Online safety groups Inclusive services
Study 21. Oueis et al. (2024). Canada. Qualitative. Exploratory qualitative survey.	Men (*N* = 206, age 18–77). Gender: trans male (*n* = 28), male (*n* = 151), genderqueer/fluid (*n* = 12), or non-binary (*n* = 11), agender (*n* = 4). Sexuality: gay (*n* = 129), bisexual (*n* = 42), questioning (*n* = 2), queer (*n* = 27), asexual/aromantic (*n* = 4), pansexual (*n* = 5)	Men who have sex with men employ several coping mechanisms. Many are open to engaging with services but perceive them to be inaccessible or to lack cultural awareness.	Avoidance, suppressing or denial Reluctance to talk about incident/impact Perpetrator concerns (guilt or fear) Rejection from support due to identity Withdrawal from shared friends Bad experiences with services Fear of being victim blamed Social pressure to move on	Desire to process the event For advice, coping skills or reassurance Routine where incidents are persistent
Study 22. Matsuzaka and Koch. (2019). United States. Qualitative. Interviews.	Sexual violence survivors (*N* = 10, age 21–57). Gender: trans female (*n* = 9), trans female /trans feminine (*n* = 1). Sexuality: pansexual (*n* = 1), queer (*n* = 2), heterosexual (*n* = 5) bisexual (*n* = 2).	Trans feminine individuals describe transmisogynistic victimization which negatively impacted their physical and mental health and self-perceptions	Feeling ostracized from the racial/ethnic groups Not feeling they will be supported Concerns about negative responses Victim blaming by authorities in relation to sex workers	
Study 23. Jackson et al. (2017). United States. Qualitative. Interviews.	Sexual minority survivors (*N* = 18, *M* age 42.2 years) who disclosed their experience. Bisexual (*n* = 3), gay (*n* = 14), or queer (*n* = 1).	Secondary victimization can significantly impact recovery.	Inability to label an incident Shame, guilt and self-blame, Stigma relating to intersectionality Lack of cultural competence in services	Receiving a positive Response to disclosure
Study 24. Scorgie et al. (2013). South Africa. Qualitative. Interviews and focus groups.	Sex workers in African countries (*N* = 136), of which 4 identified as transgender. Transgender participant age ranged from 25–34.	Sex workers exhibit unmet health needs and are frequently rejected from support services.	Being mistreated when accessing mainstream support due to being a transgender person in sex work Experiencing being mocked and invalidated in healthcare settings	
Study 25. Witcomb and Cooper (2023). England. Qualitative. Interviews.	College students (*N* = 9, age 19–24). Orientation was listed as bisexual (*n* = 2), lesbian (*n* = 4), or Queer (*n* = 3).	Participants discussed unwanted sexual attention, its impact on relationships but LGBTQ+ circles can act as a refuge.		Having LGBTQ+ friends with similar experiences Wanting advice
Study 26. Wang (2011). United States. Qualitative. Case study, interview.	The participant was a lesbian in her 50’s who had been raped in her late 20’s by a woman.	Barriers were categorized as sociocultural, difficulties in processing the incident and lack of LGBTQ+ inclusion in services.	Cultural stigma around female perpetrators Not wanting to tarnish LGBTQ+ culture Fear of being outed Fear of traumatizing others by telling their story	
Study 27. Donne et al. (2018). United States. Qualitative. Interviews	Male survivors (*N* = 34, age 21–47). Bisexual (*n* = 1) gay (*n* = 12), shifting (*n* = 1), straight (*n* = 4). One identified as male to female transgender. Focus groups involved only gay men.	Male sexual violence survivors experience unique challenges to accessing support services and engaging in professional help which can facilitate recovery.	Societal norms around male victimization Reluctance to accept or label experiences Not feeling the situation is severe enough Perceiving short-term services ineffective Inability to access inclusive support Finding support groups re-traumatizing	Having a support system Experiencing triggers Engaging with support services for other reasons Needing comfort/support
Study 28. Harris and Dunn (2019). Jamaica & United States. Qualitative. Interviews and focus groups	Men who have sex with men (*N* = 30, age 18–29) who had experienced sexual violence. Orientation: gay (*n* = 18, 60%) or bisexual (*n* = 12, 40%).	Men who have sex with men who are perceived less masculine are believed to be more vulnerable to victimization.	Fear of not being believed Shame and embarrassment Fear of being blamed Threats from the perpetrator	
Study 29. Braun et al. (2009). New Zealand. Qualitative. Interviews.	Participants (*N* = 19) were sexual violence survivors (*n* = 18) and a key informant (*n* = 1) (age 20–54). Orientation was listed as gay (*n* = 17) or bisexual (*n* = 2).	Four patterns were identified; unwanted sex involving force, consent invalidated, power dynamics and obligation to engage.	Fear of not being believed	Perceiving the experience as sexual violence
Study 30. Salter et al. (2021). Australia. Mixed methods. Survey.	Sexual minority men (*N* = 895, age 18–35). Assigned male (*n* = 848), assigned female (*n* = 41) or did not answer (*n* = 5). Gender: male (*n* = 857), non-binary (*n* = 23) or other (*n* = 10). Gay (*n* = 794).	Many disclosed an unhealthy or abusive relationship. Survivors were more likely to engage in substance use	Not being ready to disclose LGBTQ+ status and feeling one cannot disclose one without the other	
Study 31. Grocott et al. (2023). United States. Quantitative. Survey.	LGBTQ+ college students (*N* = 73) who experienced sexual violence in college (age 18–51).	Sexual minority students may disclose victimization to inform supports. Alcohol can play a role in occurrence.	Feeling ashamed/embarrassed Confidentiality/persecution concerns Perpetrator concerns, fear or guilt Lack of awareness or trust of services	
Study 32. Hequembourg et al. (2021). United States. Mixed-Methods. Interviews.	White cisgender women (*N* = 247, M age = 27.74). Orientation was listed as lesbian (*n* = 88) or bisexual (*n* = 84).	Sexual minority women reported higher rates of childhood sexual abuse but similar levels as adults compared to heterosexual people.	Being able to move on with life Wanting to forget the incident Needing to forget the incident and not talk about it	Having a strong queer support network Advocacy work reducing shame and self-doubt
Study 33. Flanders et al. (2020). United States and Canada. Mixed methods. Survey	Bisexual or plurisexual people (*N* = 245, age 18–25). Nonbinary (*n* = 159), trans man (*n* = 27), trans woman (*n* = 7), man (*n* = 4), woman (*n* = 47).	Bisexual stigma was significantly associated with sexual violence. Connecting with other LGBTQ+ survivors, was beneficial, yet many faced discrimination.	Not wanting to talk about their experience Previous experiences of discrimination Intersectionality not represented Inability to articulate the experience Rape myths leading to invalidation Fear of discrimination, not being believed	Wanting support Knowing of others with similar experiences Sense of comradery Talking with others about their journey
Study 34. Bowling et al. (2024). United States Mixed-Methods. Survey	Alt-sex individuals (*N* = 2,888, most aged 25–35). Gender: man (*n* = 898), woman (*n* = 1582), other (*n* = 392). Heterosexual (*n* = 774), bisexual (*n* = 677), pansexual (*n* = 596), gay/ lesbian (*n* = 145), asexual/other (*n* = 264)	The community generally mistrusts police and fears stigmatization and retribution from within the alt-sex community upon disclosure.	Concerns or fear for the perpetrator Fear of being outed Fear of exclusion from their community Fear reporting makes a situation worse	Desire for protection
Study 35. LaRosa et al. (2024). United States. Qualitative Interviews	Participants (*N* = 8) were transgender survivors of sexual violence (age 20–43). Some indicated sexual minority identities	Transgender survivors recovery is potentially linked with the interplay between gender dysphoria and trauma.	Withdrawal, isolation, self-blame, shame Reluctance to tarnish trans community Lack of affirming options Fear of negative reactions or losing friends	The ability to identify as a survivor A desire to advocate for others

Note. IPV = intimate partner violence; LGBTQ+ = Lesbian, Gay, Bisexual, Transgender, Queer, plus.

## Results

### Study Characteristics

Out of the 35 included studies, 30 were qualitative and involved either interviews (*n* = 25), open-text response surveys (studies 1 and 21), focus groups (study 19), or a combination of focus groups and interviews (studies 28 and 24). The quantitative study (study 31) involved a prepopulated survey with no open questions. The mixed method studies, studies 30 and 34, used online surveys with open- and closed-ended questions, study 32 used computer-assisted self-interviewing, and invited selected participants to interviews based on responses. Similarly, study 33 selected participants from an online survey to attend interviews. One paper (study 26) was a longitudinal qualitative case study conducted over a 4-year period while all others were cross-sectional. The included studies spanned from 2009 to 2025, with 14 published since 2023, evidencing the recent increase in popularity of the research topic.

The studies represent *n* = 5,543 participants, with one (study 34) accounting for more than half (*n* = 2,888). The 30 qualitative studies varied in sample sizes, with three involving large participant numbers; studies 1 and 21 involved 273 and 206 survey responses, study 24 analyzed data from 136 participants in interviews and focus groups. Study 31 involved 73 participants in their quantitative analysis. Most (*n* = 21) were conducted in the United States, with others being carried out in South Africa, Australia, Singapore, Spain, Cambodia, Portugal, Sweden, Canada, England, New Zealand or collaborations between the United States and Jamaica or the United States and Canada (as per Table 1).

Of the included studies, 10 reported results from sexual minority subgroups only (i.e., lesbian, gay, bisexual, or queer orientation), seven only reported on gender minorities, and the remaining 18 included the broader LGBTQ+ community (as per Table 1). Studies differed in their representation of other aspects of participants’ identities. Five provided minimal demographic details aside from LGBTQ+ status and did not outline additional minority identities (studies 5, 8, 15, 17, 34). The authors of study 34 described their sample as primarily white U.S. residents, aged 25–35, but provided no details relating to other participants. Study 15 did not involve participants being asked about ethnicity, while study 5 did not record such information due to the sensitive subject matter; the authors reported that this decision was taken with the intention of protecting participant anonymity. The authors of study 29 acknowledge this as a potential limitation of their research, reflecting that ethnicity may have impacted power dynamics experienced by some participants; in this case, the authors also avoided reporting such details due to concerns about participant confidentiality, and a perception on the part of the researchers that they did not possess appropriate cultural expertise to appreciate such intersecting identities. In contrast, study 19 treated race and ethnicity as aspects of participants’ identities that were central to understanding their experiences. This study recorded demographic details of the interviewee in the transcript and provided them to accompany quotes in the manuscript. While race/ethnicity was reported for the remaining 29 studies, other demographic information varied: education level was reported by 16 studies (e.g., studies 2 and 13). Six studies discussed employment (e.g., studies 12 and 22), three discussed disability status (studies 1, 2, and 35), and two religious affiliations (studies 3 and 23).

Most included studies provided some detail relating to the relationship between perpetrator and survivor. The relationship most often reported was partner (4–6, 8–11, 14, 15, 17, 19–21, 23, 25–27, and 29–33), which in some cases was further categorized as (i.e., as casual partner, romantic partner, ex-partner etc.), followed by family member (5, 8, 14, 17, 20, 28, and 32), friend (5, 8, 14, 17, 31, and 32), acquaintance (8, 12, 14, 16, 17, 20, 31, and 32), strangers (1, 14, 16, 20, 29, and 31), and sex work clients (20, 22, and 24). Five studies did not indicate the relationship between perpetrator and survivor (2, 3, 13, 27, and 35). Evidence relating to legal outcomes or alternative consequences for the perpetrators was scarce among included studies, partially because many survivors did not choose to report their victimization. However, study 21 provides one narrative of a survivor who reported a perpetrator in a position of power in a university, explaining that the perpetrator later resigned from their position. A second participant in this study described taking their assailant to court, but did not indicate any outcome in terms of conviction or sentencing.

Most of the included studies provided insight into the impact of victimization, with the most common outcomes relating to psychological distress (studies 1–9, 10–15, 17–23, 25–28, 30, 32, 33, and 35) with some specifying that this took the form of PTSD (studies 1, 19–22, 26, 33, 35) and suicidality (studies 1, 3, 8, 14, 17, 18, 20–22, and 35). In terms of coping mechanisms, a number of studies described substance use (e.g., studies 3, 17, 20–22, 27, and 32) or withdrawal from relationships including social, sexual and romantic (e.g., studies 5, 8, 12, 14, 17, 20–22, 25–28, and 35). Studies 9, 16, 29, 31, and 34 did not include any detail on the impact of sexual violence. Study 14 documented several instances where experiences of victimization continued to exert an influence over survivors’ lives, sometimes long after the abuse had ended. This is illustrated by one survivor’s comment “if it hadn’t happened, maybe I would be a different person today.” However, survivors reacted and processed their experienced in many different ways. This is evidenced by Study 21, which reports the experiences of a bisexual man who explained that although he did not want to minimize his victimization experiences, he had “shrugged them off.” In contrast, a gay participant described feeling deeply affected, struggling to cope, and experiencing depression and suicidal thoughts as a result. Another gay man shared, “it follows me every day,” emphasizing the lasting impact of his experience.

### Quality Appraisal

The quantitative study (study 31) scored 20 points out of 27, using the Quality Review and Critical Appraisal method (Dunne et al., 2017) indicating good quality. Mixed methods and qualitative studies were reviewed by MMAT (Hong et al., 2018) and CASP tool (CASP, 2024) respectively. These tools discourage assigning an overall score, suggesting instead that the text is considered as a whole. In each category, included studies were ranked as high quality, however with some potential limitations. The MMAT and CASP tools prompt evaluating the suitability of the recruitment strategy to address the research question. While most focused recruitment on LGBTQ+ groups, some expanded efforts to wider networks. Study 21 utilized groups which support sexual minority men, while study 33 combined convenience sampling distributing flyers online and in public spaces. The former approach may limit participation from individuals who are not publicly or openly affiliated with the LGBTQ+ community. Additionally, although engaging in reflection about one’s positionality is considered best practice according to the Consolidate criteria for reporting qualitative research (COREQ) checklist, 10 studies fail to report such practice (e.g., studies 8, 19, and 27). Milner (2007) suggests that this is particularly important when working with minority groups. The CASP tool also queries how ethical considerations were approached. Detail on such considerations was inconsistently reported; only 12 studies described support offered to participants, typically involving a list of support services (e.g., study 35).

### Data Charting and Synthesis

Of the 35 included studies, 21 contained both barriers and facilitators to help-seeking for LGBTQ+ sexual violence survivors (studies 2, 6–8, 10–13, 16–21, 23, 27, 29, and 32–35), 12 contained barriers only (studies 1, 3–5, 8, 14, 22, 24, 26, 28, 30, and 31) and 2 contained facilitators only (studies 15 and 25), a summary of these findings is presented in Table 1. In 25 studies, it was possible to determine which LGBTQ+ subgroup the barrier or facilitator pertained to (studies 1, 2, 4, 6–15, 18, 20–27, and 33–35), either because the study involved only one subgroup or because participants’ identities accompanied quotes. A narrative synthesis of the review findings is presented below. Barriers and facilitators will be outlined here individually for ease of analysis, but in reality, many of these factors are intertwined and serve to amplify one another.

#### Barriers

##### Discrimination Experiences

The included studies present numerous accounts of LGBTQ+ survivors encountering homophobia and/or transphobia on their recovery journey (e.g., studies 1, 2, 4–6, 8–11, 15–30, and 32–35). In some cases, participants described navigating these issues from childhood. For example, in study 18, several participants revealed that they had grown up with parents who were unsupportive of their sexuality and/or gender identity, which led to almost all participants choosing to withhold their victimization from their families. Many of those who disclosed victimization reported negative outcomes, which enhanced emotional distress. Similarly, study 33 presents one survivor’s perspective that their school’s lack of support for their gender identity was a barrier for them in reporting victimization. Some acts of discrimination experienced by participants were not explicitly homophobic or transphobic but instead reflected systemic biases in a heteronormative and cisnormative society. For example, in one qualitative study from the United States, (study 2) a participant referred a lack of awareness in services around providing care to transgender people, reporting that assuming gender, confusion about pronouns and dead-naming service users (referring to a person by their birth name when they have changed their name to match their true gender identity) was common in their experience. Some studies described transgender survivors facing additional barriers when seeking emergency accommodation. One example is from study 4, where a transgender woman was sexually victimized by another resident and upon reporting this to a member of staff was advised that it was “in their best interest” to transfer, because these incidents would be expected to happen to someone who presented as female. This participant reported feeling invalidated and silenced by the experience. Also, in study 4, a transgender Latina woman described how staff in a homeless shelter told her she wouldn’t be safe there, as the center was full of men. When she turned to a women’s homeless shelter, they rejected her as she was not born female. Although she was later accepted by a women’s shelter, she continued to endure transphobic comments from staff as well as staring and unkindness from other residents. Many participants described an avoidance and mistrust of the police (e.g., studies 8, 9, 16, and 20). Homophobic attitudes were conveyed to survivors in a number of ways, as highlighted by participants in study 9 who described police interactions as involving “overt condemnation” or comments that constituted re-victimization. For example, one participant reported being told that “you can’t rape the willing” upon reporting their experience to the police. Within this study, all participants of color reported that they had been racially profiled by police, that they expected unhelpful and disrespectful responses to reporting, and that they had an acute awareness of their heightened potential for discrimination based on their identities as Black LGBTQ+ people. Past experiences of discrimination based on race (e.g., studies 9 and 16) or sexuality (e.g., studies 16 and 21) were seen to contribute to survivors seeing support services as inaccessible to them.

Many participants reported feeling that services would be unable or unwilling to help them (e.g., studies 2, 6, 17, 19, and 35). This was often more pronounced for those with multiple marginalized identities (e.g., studies 12, 27, 20, 21, and 26). LGBTQ+ people with additional marginalized identities described feeling ostracized from multiple communities due to experiencing additional types of discrimination besides homophobia and transphobia, such as racism or ableism. For example, one study (study 35) details how, as a physically disabled transgender man, help-seeking options were limited. Another account (study 4) involved a participant explaining that as a transexual female, an immigrant, and a person engaging in illegal activity (hormone sharing) involving the police would have worsened their situation. Many survivors disengaged from services due to experiencing discrimination based on identity or inability to adhere to gender expectations (studies 1, 4, 10, 16, 17, 19, 20, 22, 24, and 35). One bisexual man (study 1) described being sexually harassed for not meeting stereotypical masculine expectations, while help and recognition is inaccessible because of his sex, explaining “there is no winning in this scenario.” This was echoed by another participant (study 23) who shared that racism in the LGBTQ+ community and bi-phobia in the Black community result in them never feeling completely safe in either space, they explained “when all of my identities intersect, there is always something that I am still hiding, fighting, or I am not 100% there in any of them.”

##### Social Endorsement of Rape Myths and Stereotypes

Many survivors who overcame internal barriers to acknowledge victimization reported encountering social skepticism, which distorted their experiences and discouraged help-seeking. This includes rape myths (for example the idea that rape must involve physical force and violence) and cultural expectations of “real” victims. Participants described how mainstream narratives around these concepts often fail to account for LGBTQ+ survivors, further contributing to internalized doubt. For example, included studies document instances of survivors being told that their experience was due to the way they dressed (study 10) or how much alcohol they had consumed (study 11). Survivors described how these responses inhibited future disclosures and help-seeking attempts (e.g., studies 11, 17, and 18). Participants in study 29 explored how the LGBTQ+ community can also perpetuate these narratives, such as the stereotype that gay men exist in a perpetual state of wanting sex, or that a person can be owed sex in certain contexts. For others, a fear of reinforcing negative stereotypes about LGBTQ+ culture was itself a barrier to disclosure (e.g., studies 18, 23, 26, and 35). Some avoided seeking help due to not wanting to cause harm to their perpetrator (studies 6, 14, 19, 21, 31, and 34) or fearing retribution (studies 16, 19, 21, 28, 31, 34, and 35) as well as more general negative consequences. A lack of trust in the legal system, and in particular, members of the police, was cited in a number of studies. One participant (study 21) described ongoing guilt about reporting their victimization, expressing mistrust of the legal system and concern that, by coming forward, they may have caused more harm to the perpetrator than they themselves experienced. Their description of how this decision “weighed on them” over the years demonstrates the added burden of survivorship that can sometimes come with reporting. Beyond individual experiences of stigma, survivors are impacted by their efforts to navigate the cisheteronormative cultures within services.

##### Feelings Associated With Shame and Self-Blame

Many survivors shared barriers relating to feelings of shame, self-blame, and embarrassment (studies 1, 7, 8, 12, 19, 26, 28, 31, and 35). One study (study 28) reports that many of their participants blamed themselves for giving their power to their abuser or felt they had caused their victimization in some way. Another participant (study 25) described feeling that being gay, and presenting as a gay woman in society, led to them being a target for perpetrators of sexual violence. This reinforced internalized homophobia, sexuality-related shame, and a sense of being “othered.” Survivors often feared that they would receive these messages from people they disclosed their experiences to. Specifically, participants feared that they would be blamed for the incident (studies 5, 16, 18, 20, 21, 28, and 35) or disbelieved (studies 6, 8, 20, 23, 28, 29, and 35). Unfortunately, the experiences of those who did disclose victimization indicate that such concerns are valid, evidenced by survivors’ accounts of how disclosing and reporting sexual violence can lead to losing friends, damage relationships with family members and even result in dismissal from employment (study 23). Concerns about invalidation of experiences were also prominent (studies 4, 5, 18, and 35) as well as fears of being mistreated (studies 6, 16, and 35) by both formal services (particularly police) and informal supports. Participants expressed particular fear and distrust regarding the police. For example, in study 22, a participant who identified as a transgender sex worker was sexually assaulted by a patron, and was told by the attending detective “I don’t blame the guy. I would have had the same reaction. You knew what you were getting into” before concluding that no crime had taken place. One participant (study 23) likened these negative responses to being re-victimized. Two studies (studies 23 and 27) reported that male victims may experience heightened fear that stereotypes will result in their rejection from supports. In addition, negative responses to disclosure can lead to survivors questioning the accuracy of their interpretation of their experience, as expressed by a participant in study 23.

##### Rejection of the Experience

Many survivors described reacting to their experiences through denial, minimization, downplaying, and normalizing the experience (studies 1, 11, 14, 17, 27, and 31). This was often linked with an inability to label the experience as violence (studies 5, 19, and 23), or to integrate victimization into one’s identity (studies 1, 4, 17, and 23). In addition, several studies made reference to the normalization of sexual violence (e.g., studies 1, 2, 4–12, and 15–35). For example, one participant (study 17) described believing it was a woman’s “duty” to have sex with men, while another (study 26) reported rejecting the very possibility of a female perpetrator, as this conflicted with their mental construction of the “lesbian utopia.” Finally, a survivor of childhood sexual abuse (study 14) recalled assuming that the experience of abuse was commonplace and happened to many children. While these barriers can create significant obstacles to seeking help, some factors have been identified that support and facilitate LGBTQ+ survivors in accessing support.

#### Facilitators

##### Need to Connect With Others

In many included studies, survivors described an intrinsic need to engage in help-seeking to gain support and connect with others (e.g., studies 11, 16, 21, and 27). Survivors who had been able to process and understand their experiences sometimes reported becoming more comfortable engaging with help-seeking over time (e.g., studies 7 and 19). One study (study 13) suggested that this may be because survivors are “shell shocked” in the immediate aftermath, and that denial can act as a protective mechanism which helps them maintain their normal level of functioning. Survivors wanted different things from services and supports; one survivor (study 33) explored the value in comradery when survivors with shared experiences can support each other and discuss coping mechanisms and the mental health services which they have engaged. Some reported wanting advice (studies 21, 23, and 25). Others wanted help in moving away from unhelpful coping strategies such as avoidance and substance use (studies 19, 20, 21, 27, and 33). Many contacted services seeking protection (studies 8–10, 16, and 34) or to pursue legal justice (studies 10 and 16). Finally, some reported feeling compelled to channel their anger into productivity, and to incite social change by encouraging others to speak out (studies 7 and 35).

##### Social Encouragement and Empowerment

Many survivors were encouraged to seek formal support by friends or other survivors. In study 16, a participant described their family as very supportive, detailing how their family encouraged them to report the experience to police. Several participant accounts highlighted the role of community, and explained how knowing others with similar experiences supported help-seeking (studies 20, 25, 33, 35). Others described how having existing support systems, formal or informal, made help-seeking less daunting (studies 20, 21, and 27). Findings from studies 7 and 23 suggest that positive disclosure responses allow survivors to become more comfortable exploring their own story and sharing it with others. For example, a participant in study 7 described the sharing of their story, after years of keeping it to themselves, as an empowering act which allowed them to regain feelings of control. One participant described how talking about her experience, and speaking with other women, changed her view of the experience, addressing her barriers of self-blame and the idea that she was “not the right kind of survivor to get to talk about it.” Another participant in the same study explained how sharing her story diminished the power it once held over her. Positive reactions from informal supports (e.g., from friends, families, etc.) sometimes helped survivors gain the confidence they needed to engage professional support.

##### Positive Engagement With Support Providers

Some survivors reported positive experiences with service providers, which supported continued engagement with help-seeking. In two studies (studies 2 and 27), survivors initially engaged mental health support organizations for alternative reasons and found that building trust over time enabled them to later disclose victimization. Individuals who hold marginalized minority identities seeing people they perceive as “like them” within services emerged as a powerful facilitator. In study 2, one survivor shared that, for them, connecting was easier in an affinity group for people of color, as they felt better understood. This was echoed by a second participant, who discussed their preference for a therapist who understood Latinx culture. Study 20 reported how sex workers praised an online safety group as a source of community, in connecting them with others who could understand their unique situation and where they felt safe from judgment. An ability to demonstrate understanding and acceptance of the survivor’s gender, racial, and sexual identities was seen as highly important (e.g., studies 17, 19, and 20). Numerous studies referenced the impact of service providers who empowered survivors and made them feel deserving of support and kindness. This generally involved providers who cultivated a safe space, where survivors were believed, understood and encouraged (e.g., studies 15, 17, 19, and 20). Such spaces were seen to validate survivor’s experiences as well as their subsequent reactions to sexual violence.

## Discussion

This review aimed to analyze the factors that act as barriers and facilitators to help-seeking for LGBTQ+ people who have experienced sexual violence. A summary of the findings can be reviewed in Table 2. The included articles indicate a strong intrinsic need to engage in help-seeking to gain support, validation, understanding, or connection in the aftermath of sexual violence. They highlight the negative impacts of victimization, socially, relationally, and psychologically, which are consistent with the wider literature on victimization (e.g., Geppert et al., 2024). Unfortunately, our findings suggest that despite the drive and desire to seek help, survivors who reach out for support post victimization are often met with barriers which overlap and are mutually reinforcing.

**Table 2.** Summary of Critical Findings.

Barriers to help-seeking	• Cisnormative and heteronormative systems (experiencing either a lack of cultural awareness or discrimination, or a belief that they will experience this upon engagement) • Feelings of shame, self-blame, and embarrassment • Fear of negative responses to disclosure (blaming, disbelief, invalidation, mistreatment) • Denial, minimization, and normalizing the experience • Inability to label their experience as sexual violence or to consider themselves a victim • Social endorsement of rape myths and stereotypes • Fear of tarnishing LGBTQ+ identity, or the wider LGBTQ+ community • Concerns relating to the perpetrator (not wanting to cause harm, or fearing retribution and negative consequences • Mistrust of service providers (most notably, mistrust in police and the legal system)
Facilitators to help-seeking	• Intrinsic need to connect with others • Recognizing and being able to label sexual violence • Connecting with other survivors and sharing stories with them • Positive disclosure responses • Inability to cope alone • Practical considerations (for protection or in pursuit of legal justice) • Desire to channel the experience into positive change • Encouragement from friends and family • Positive interactions with service providers • Feeling service providers understand their identity • Seeing people within services they view as similar to themselves • Feeling listened to, validated, supported, and believed
Important considerations	• Individuals who hold multiple minority identities may experience addition barriers to help-seeking and higher levels of discrimination and require special consideration in service design and implementation • The Power Threat Meaning Framework can be utilized to understand how the factors which impact help-seeking are interconnected. It can aid in explaining how experiences of discrimination, a diminished sense of one’s abilities and self-worth and a view that services, are ineffective or harmful restrict engagement with help-seeking opportunities and reinforce barriers to support. • Our review highlights evidential gaps given the limitations of our findings. We suggest that more quantitative, longitudinal research is needed, involving individuals who hold multiple minority identities

*Note*. LGBTQ+ = Lesbian, Gay, Bisexual, Transgender, Queer, plus.

In some instances, survivors believed that they were victimized because of their LGBTQ+ identity, which reinforced feelings of shame, self-blame, and internalized homophobia or transphobia. Self-blame is common among survivors of all sexualities (e.g., Bhuptani & Messman-Moore, 2019), however, LGBTQ+ people may experience these feelings more acutely and in unique ways relating to their identities. Binion and Gray (2020) suggest that internalized homophobia can exacerbate feelings of shame and create a cognitive distortion where survivors feel they do not qualify as a “real” victim deserving of support. In study 2, a survivor explained that she had waited a year on the campus-based support services waitlist, when she was finally offered a place, she felt guilty that her inclusion was depriving someone else. Many participants reported feeling helpless and as though they had no options for support (e.g., studies 4, 8, and 16).

Experiences of discrimination, understood through the lens of the PTMF, may explain why many survivors come to view support options as ineffective or even as threatening, and how these perceptions act as barriers to help-seeking. Many survivors expressed the view that reporting victimization was futile (e.g., studies 8 and 14). The experiencing of services as a threat was also a common theme throughout the included studies. Even in the absence overt homophobia and discrimination, LGBTQ+ people face structural barriers when seeking support. Butterby and Donovan (2023) conducted interviews with police and LGBTQ+ domestic abuse survivors in the United Kingdom. They found that police believed treating all cases the same, regardless of victim identity, results in an equitable service for all. However, survivors explained how the established processes draw upon heteronormative stereotypes, which can lead to LGBTQ+ survivors feeling misunderstood and minimized. Hodge and Sexton (2018) found that many LGBTQ+ people hold negative perceptions of police, with most (74.8%) reporting that they believed being an LGBTQ+ victim of crime would negatively impact how they are treated. These perceptions were even more pronounced among racial minorities and transgender participants, aligning with the findings of this review. One participant from this study commented, “I think law enforcement is pretty good for white gay cis men. I think it can be terrible to trans people, especially trans women of colour.” The participant went on to explain their view that the culture of machismo among police departments fosters negative attitudes toward anything that challenges traditional gender norms or masculinity, in particular things that are perceived as queer.

Although this review focused on experiences relating to sexual and gender minority identities, the results also highlight the importance of intersectional awareness (e.g., studies 1, 4, 6, 18–20, 22–27, 33–35). For example, study 22 reported how transgender racial/ethnic minority participants saw their intersecting identities as relegating them to “the bottom of the barrel of what’s not accepted.” Study 9 reported that all 12 participants of color expressed police distrust, one shared that they couldn’t always determine the role of racism or homophobia in discrimination, stating, “it’s just something I know in my bones—like I can tell when it’s more about one of those things than the other, but it’s usually about both”. This mirrors the narrative of study 35, whereby all 8 participants discussed the unique stressors associated with intersecting marginalized identities. One, a Black, queer, nonbinary person, reflected that the microaggressions and mistreatment they experienced could be driven by multiple aspects of their identity, making it hard to pinpoint the exact motives. Another described struggling to find a physician to manage chronic pain resulting from victimization due to their identity as a physically disabled trans man. Our findings suggest survivors who experience intersecting forms of oppression due to holding multiple marginalized identities may be at increased risk of experiencing discrimination.

The findings also highlight gender-related difficulties and barriers in to help-seeking. Many male, non-binary, and transgender participants found accepting victimization difficult, and perceived that friends and support systems would reject them if they disclosed their experiences (studies 4, 9, 20–23, 27, 28). Many engaged in denial and minimizing both the incident and its power over them (e.g., study 5). This aligns with research on cis male victimization, that societal expectations of masculinity can inhibit men from identifying as a victim (McAulay, 2024). Several participants cited rape myths about victims being weak and submissive (e.g., studies 5 and 23), or societal stereotypes of masculinity that define “real men” as strong and capable of defending themselves (study 27). Crenshaw (1989) suggests that when the societal expectations of one identity conflict with another, the individual may exhibit personal, emotional and relationship problems. Two studies (studies 19 and 23) suggest that LGBTQ+ people may be more vulnerable to rape myths, as they often lack social scripts for what healthy relationships or abusive behavior look like.

Bowleg et al. (2023) suggested that understanding individuals with multiple marginalized identities involves exploring how systems of power and oppression, including race, occupation, sexuality, and gender impact their lived experience. While many studies did include racial and ethnic demographics, other details, such as occupation/employment status, disability status, religious affiliation, education level attained, substance use history, mental health diagnosis, and socioeconomic status were often absent. Forcing individuals to filter their experience through a single-axis analysis denies all other sources of stigma, oppression, and discrimination, which often results in the de-prioritization of their unique needs and inhibiting their ability to access support (Bowleg, 2008; Crenshaw, 1989; Combahee River Collective, 1977). Inadequate acknowledgment of intersecting identities among participants is not the only form of erasure; in some cases, researchers exclude multiply marginalized participants entirely (Bowleg et al., 2023). For example, study 32 excluded transgender and gender non-conforming women due to practical challenges and insufficient conceptual tools. Our results are convergent with Bowleg et al.’s (2023) findings that despite growing awareness, intersectionality remains under-considered in LGBTQ+ research. This can result in the most disadvantaged members of social groups being further marginalized, and the needs of a “default service users” being prioritized. This is not merely detrimental to research, policy, and service development, but it also has the potential to directly influence survivors themselves, and to shape how they perceive their victimization.

Despite the barriers presented, our results identified several facilitators to help-seeking, which could be used to improve service provision. The most significant relates to the levels of competency and compassion of those whom the survivor chooses to engage with. Positive experiences reported by survivors aligned strongly with the six key principles of a trauma-informed approach (safety, trustworthiness and transparency, peer support, collaboration and mutuality, empowerment voice and choice, and cultural issues), as proposed by the Substance Abuse and Mental Health Services Administration (2014). Many survivors in the included studies described slowly accepting their victimization, which is common among all survivors; Lanthier et al. (2018) suggested that most disclosures occur more than 7 days after an incident, with many waiting months or years. Among those who did engage with help-seeking, many praised service providers’ ability to cultivate a safe space. Study 7 presented multiple accounts of survivors who felt that sharing their story in their own words helped them to regain their sense of power and control. Herman (2015) observed that disempowerment and disconnection are core experiences of psychological trauma, while empowerment, the restoration of autonomy, and the rebuilding of trust in others are necessary for recovery. Conversely, study 35 reported on two participants who had engaged with non-affirming therapists with inadequate understanding of their transgender identities, leading to complications within the therapeutic relationship. Several participants highlighted the importance of therapists who are skilled in talking about trauma gradually, in a way that is safe for the survivor (studies 15 and 19), and avoiding projecting their own beliefs. This again aligns with Herman (2015) who posed that therapists should serve as assistants for survivors, promoting their autonomy, validating their experiences and collaborating on healing efforts.

There is no one factor that was directly tied to feelings of safety; some describe a support worker being LGBTQ+ or the same ethnicity, while others showed no preference on the therapist’s identity, provided that they felt validated (e.g., studies 17 and 20). These results outline how people resist engagement when they fear being misunderstood, disbelieved, or discriminated against. Yet, when they feel cared for, and are met with positive regard, survivors willingly engage with supports. Survivors sought support in various forms; many referenced a need to connect with other survivors (study 33), and in some cases LGBTQ+ survivors (study 25) and/or people of the same ethnicity. Study 2 reported that for college survivors peer-led support was considered crucial, particularly by those of color, reinforcing a need for intersectional awareness. These findings have several implications for researchers, practitioners, and policymakers (summarized in Table 3).

**Table 3.** Implications for Practice, Policy, and Research.

Implications for Practice	Implications for Policy	Implications for Research
Ensure staff can provide trauma-informed care which integrates minority stress and intersectionality frameworks	Ensure supports are available for intersectional service users and adequately funded	Greater representation of those with multiple minority identities
Facilitate the development and operation of peer-led support services and collaborative partnerships with minority groups	Meaningful collaboration with minority services	Production of more quantitative and/or longitudinal research
Co-design material with minority support groups	Raise awareness, refuting stereotypes & myths and informing the public on disclosure responses	Increased focus on strengths and ongoing interventions within the LGBTQ+ community

*Note*. LGBTQ+ = Lesbian, Gay, Bisexual, Transgender, Queer, plus.

### Implications

#### Implications for Practice

This review demonstrates a need for services to provide trauma-informed support that integrates minority stress and intersectionality frameworks. Our finding that many survivors are better able to engage when different identities are represented within services suggests an urgent need for support providers to engage in meaningful collaboration and co-design with marginalized minority groups, and to ensure that they are hiring diverse teams. This can facilitate a deeper understanding of the barriers these groups face and lead to the improvement of service delivery. This diversity can help enable support providers to identify and eradicate discrimination based on factors such as homophobia, transphobia, racism, and ableism from their practice. These efforts should include practitioners facilitating the development and operation of peer-led support services for survivors to connect with each other and share their stories in this setting. In line with evidence from this review, providers should incorporate an understanding of mental health in its social context. The PTMF and minority stress theory can be used to educate staff on the specific needs of LGBTQ+ service users and prevent services from inadvertently perpetuating bias and microaggressions. However, as evidenced by our review existing research remains in its infancy and is largely based on U.S. studies. As such, the lived expertise of individuals from marginalized communities often surpasses what the current evidence base can offer, underscoring the need to prioritize their voices in shaping inclusive and effective services.

#### Implications for Policy

Policymakers should enable support organizations to allocate funding toward meeting the specific needs of service users with marginalized identities, particularly those who hold multiple marginalized identities. Furthermore, they should encourage meaningful collaboration with minority services and advocacy organizations when designing policies that affect those groups. Considering our findings that many survivors have difficulty in recognizing sexual violence, or considering themselves as deserving of support, policymakers should facilitate the development of large-scale awareness campaigns which refute myths and promote the identification and labeling of sexual violence. Participants in study 1 called for their personal experiences to be represented in educational programming to better represent the “true dynamics” of such situations. One such campaign, described by Oesterle and Eckhardt (2021) focused on unique forms of sexual violence experienced by LGBTQ+ people, grounded in Minority Stress theory. Participants reported a greater awareness of sexual violence in the LGBTQ+ community and a greater appreciation for the prevalence of victimization after exposure to the campaign. Campaigns should be designed with awareness too of the fact that many survivors first disclose experiences to informal supports and should spread awareness about the power that disclosure responses have to suppress or encourage help-seeking as well as provide guidance on positive responding. Such campaigns should be designed in collaboration with minority services to increase trust and engagement. Irvine-Collins et al. (2023) explored the effects of a social marketing campaign on providing positive disclosure responses to survivors. Although this study was not specific to LGBTQ+ populations (demographic details on sexuality gender identity and sexual orientation were collected but not reported), the findings indicate that this may be a positive avenue. This evaluation reported that social marketing campaigns in university settings were useful for supporting students in understanding how to provide positive responses to disclosures of sexual violence, and in raising awareness of university-based supports. This indicates a potential role for such campaigns in addressing barriers to support seeking in LGBTQ+ communities.

#### Research Implications

The included studies were primarily qualitative and cross-sectional. Future research should incorporate quantitative and/or longitudinal methodologies, which could capture changes over time and provide more comprehensive insights. Researchers should conduct studies, where possible, through an intersectional lens, by allowing participants to self-identify (Bowleg, 2008) or providing a wide range of identification options where self-identification is not possible. This could support the inclusion of underrepresented identities, such as LGBTQ+ people who are also marginalized or minoritized in other ways. Finally, this review encountered more barriers than facilitators to help-seeking, demonstrating a focus on perceived weaknesses when researching the LGBTQ+ community. Future researchers should attempt to explore strengths within this community so that they can be maximized and leveraged. Bowleg (2021) argues that research, without action, cannot address health inequalities. Mustanski and Macapagal (2023) suggest that this holds true for the LGBTQ+ community, as despite the surge in LGBTQ+ research over the past decade, few studies have transcended beyond documenting disparities and utilized existing knowledge to assess the efficacy of evidence-based interventions. Research on LGBTQ+ mental health typically focuses on perceived weaknesses and vulnerabilities, meaning that strengths are overlooked, or not understood. For example, in a scoping review on strength-based approaches to LGBTQ health research by Colpitts and Gahagan (2016), the authors identified resilience as a conceptual framework. However, they reported that the unique experiences of LGBTQ+, in particular relating to discrimination, may contribute to resilience in a unique way, or that LGBTQ+ people may have unique resilience factors, which could be utilized to promote positive mental health. In building upon this, Colpitts and Gahagan suggest that interventions that focus on promoting resilience and incorporating an intersectional lens may be useful in health promotion strategies. Bowleg and Bauer (2016) caution that awareness of intersectionality requires an understanding beyond categorization of social identities, incorporating an understanding of social positions, group-level effects, and relevant environmental variables. They caution that power and privilege is a core construct of intersectionality and should be a primary consideration when working with people who hold multiple marginalized identities. In addition, Bowleg (2021) highlights the efficacy of grassroots initiatives, with community-based leadership in addressing barriers to health equality. Within the included studies, some participants described factors which supported their healing, for example, in study 33, a survivor described the “miraculous” impact of cognitive processing therapy “it was just like suddenly my world changed and it didn’t seem dangerous anymore (. . .) the hypervigilance was gone. The guilt was gone. I just like, changed my perspective about what happened.” Future researchers should explore survivor-centered recovery pathways for LGBTQ+ people. In so doing, they may gain valuable insight on effective techniques for supporting recovery and, through collaborative efforts with practitioners, could develop evidence-based interventions to support recovery for this population.

## Limitations

This review has compiled existing evidence relating to barriers and facilitators to help-seeking for LGBTQ+ survivors of sexual violence, albeit with some limitations. As previously noted, only one of the included texts was longitudinal, and most were qualitative and involved relatively small numbers of participants. In addition, the vast majority of studies were conducted in Western contexts, primarily the United States. The exclusion of studies involving participants under 18 means that these results apply to adult LGBTQ+ survivors only. The recruitment procedures may also mean individuals who are not able to be open about their LGBTQ+ identities may be underrepresented. So too, the different ways sexual violence is categorized in various studies should be considered, for example most described sexual violence as “unwanted sexual content” (e.g., studies 5 and 17). Another (study 9) promoted recruitment flyers stating, “have you experienced rape or sexual assault?” Study 1 asked participants a series of behavioral-based items to verify victimization. These differences may mean findings are not directly comparable, especially given the prevalence of denial and minimization. Finally, despite our attempts to be inclusive of all LGBTQ+ subgroups, some are underrepresented in our dataset (including intersex people). Future research should attempt to add to available literature by incorporating the experiences of these underrepresented groups.

## Conclusion

This review has presented a narrative summary on the complex interconnected factors, which impact help-seeking journey of LGBTQ+ people who experience sexual violence. Despite a need to seek support, many find that their efforts inhibited by barriers relating to marginalization and discrimination. Our results suggest that feelings of shame, self-blame, fear of disclosure, rejection of the experience, and victim identity can all inhibit help-seeking. Experiences of discrimination, whether direct, vicarious, or anticipated, were found to contribute to withdrawal or disengagement, even for those who had sought help. Such experiences were commonly reported among our included studies, with participants citing the police as particularly LGBTQ+phobic. The findings highlight the inequalities faced by LGBTQ+ survivors, who risk being misunderstood in a cisheteronormative context, where discrimination is a common response to seeking support from both formal and informal sources. In the absence of overt discrimination, a lack of cultural awareness was found to inhibit engagement with formal supports. In contrast, the desire to connect with others, social encouragement, and empowerment were identified as powerful facilitators of help-seeking. Additionally, support workers’ abilities to create safe environments and demonstrate understanding was crucial to maintaining engagement. The findings emphasize the importance of intersectional awareness within support services. We argue that the PTMF can complement such an approach, providing a lens to understand the social contexts that LGBTQ+ survivors of sexual violence navigate. This framework may support the development of evidence-based, strength-focused interventions that overcome barriers faced by members of marginalized groups. Such interventions could offer competent, affirming and trauma-informed care, which support not only LGBTQ+ survivors of sexual violence, but all survivors who hold marginalized identities, who might otherwise face heightened risks of marginalization, discrimination, or exclusion.

## Supplemental Material

sj-docx-1-tva-10.1177_15248380251355902 – Supplemental material for Barriers and Facilitators of Help-Seeking for LGBTQ+ Survivors of Sexual Violence: A Systematic Review Supplemental material, sj-docx-1-tva-10.1177_15248380251355902 for Barriers and Facilitators of Help-Seeking for LGBTQ+ Survivors of Sexual Violence: A Systematic Review by Ciara Buckley, Aiswarya Radhakrishnan, Lorraine Boran, Maggie Brennan and Áine Travers in Trauma, Violence, & Abuse
