# Supplementary material for: Barriers and Facilitators of Help-Seeking for LGBTQ+ Survivors of Sexual Violence: A Systematic Review
Source: Trauma Violence Abuse. 2025 Aug 17;27(4):1565–83. doi: 10.1177/15248380251355902 (PMC13578651; doi:10.1177/15248380251355902)
Supplement: Supplementary file 1 [file sj-docx-1-tva-10.1177_15248380251355902.docx]

**Supplementary File**

**Appendix A: Search terms used with each database.**

Key concepts: LGBTQ+, sexual violence and help-seeking. Databases: CINAHL, MEDLINE, PsycINFO and Web of Science.

**CINHAL**

**LGBTQ+.** LGBTQ+ OR LGB* OR “Non-heterosex*” OR “Non-monosex*” OR “Sexual minori*” OR SGM OR Bisexual OR Pansexual OR Queer OR MSM OR “Men who have sex with men” OR WSW OR “Women who have sex with women” OR “Diverse Sex*” OR “Same*Sex” OR “Sexual and gender minorit*” OR Gay OR Lesbian OR Asexual OR “Gender fluid*” OR Genderqueer OR “Gender nonconform*” OR “Non-binar*” OR Trans* OR “Gender minorit*” OR “Gender varian*” OR Homosex* OR GLB* OR nonbinary OR Agender OR Aromantic OR Bigender OR Demigender OR Demisexual OR Multisexual OR “Third gender” OR “Two*spirit” OR (MH "LGBTQ+ Persons+") OR (MH "Sexual and Gender Minorities+") OR (MH "Gender-Nonconforming Persons+")

AND

**Sexual violence.** “Sexual violence” OR “sexual coercion” OR “sexual assault” OR Rape OR “Sexual Abuse” OR “Image W3 abuse” OR “Sexual Harassment” OR “Child* sexual abuse” OR “Sexual *victimization” OR “Incapacitated assault” OR “sexual* violen*” OR “date rape” OR “intimate partner violence” OR “partner violence” OR IPV OR “partner abus*” OR “dating violence”

AND

**Help-seeking.** “Help seeking” OR “Seeking help” OR “Information seeking” OR “Assistance seeking” OR support OR “Mental health” OR “Service access" OR “Service use*” OR “Social support” OR reporting OR police OR hotline OR Disclos* OR Counselling OR Therapy OR “self-help” OR “support groups” OR “care seeking” OR (MH "Help Seeking Behavior") OR (MH "Support Groups")

**MEDLINE**

**LGBTQ+.** LGBTQ+ OR LGB* OR “Non-heterosex*” OR “Non-monosex*” OR “Sexual minori*” OR SGM OR Bisexual OR Pansexual OR Queer OR MSM OR “Men who have sex with men” OR WSW OR “Women who have sex with women” OR “Diverse Sex*” OR “Same*Sex” OR “Sexual and gender minorit*” OR Gay* OR Lesbian* OR Asexual* OR “Gender fluid*” OR Genderqueer OR “Gender nonconform*” OR “Non-binar*” OR Trans* OR “Gender minorit*” OR “Gender varian*” OR Homosex* OR GLB* OR nonbinary OR Agender OR Aromantic OR Bigender OR Demigender OR Demisexual OR Multisexual OR “Third gender” OR “Two*spirit” OR (MH "Sexual and Gender Minorities") OR (MH "Transgender Persons") OR (MH "Gender-Nonconforming Persons") OR (MH "Intersex Persons") OR “women who have sex with women”, OR “men who have sex with men”

AND

**Sexual violence.** “Sexual violence” OR “sexual coercion” OR “sexual assault” OR Rape OR “Sexual Abuse” OR “Image W3 abuse” OR “Sexual Harassment” OR “Child* sexual abuse” OR “Sexual *victimization” OR “Incapacitated assault” OR “sexual* violen*” OR “date rape” OR “intimate partner violence” OR “partner violence” OR IPV OR “partner abus*” OR “dating violence” OR (MH "Sexual Harassment") OR (MH "Sexual Trauma")

AND

**Help-seeking.** “Help seeking” OR “Seeking help” OR “Information seeking” OR “Assistance seeking” OR support OR “Mental health” OR “Service access" OR “Service use*” OR “Social support” OR reporting OR police OR hotline OR Disclos* OR Counselling OR Therapy OR “self-help” OR “support groups” OR “care seeking” OR (MH "Help-Seeking Behavior") OR (MH "Information Seeking Behavior") OR (MH "Psychosocial Support Systems") OR (MH "Social Support")

**PsycINFO**

**LGBTQ+.** LGBTQ+ OR LGB* OR “Non-heterosex*” OR “Non-monosex*” OR “Sexual minori*” OR SGM OR Bisexual* OR Pansexual* OR Queer* OR MSM OR “Men who have sex with men” OR WSW OR “Women who have sex with women” OR “Diverse Sex*” OR “Same*Sex” OR “Sexual and gender minorit*” OR Gay* OR Lesbian* OR Asexual* OR “Gender fluid*” OR Genderqueer OR “Gender nonconform*” OR “Non-binar*” OR Trans* OR “Gender minorit*” OR “Gender varian*” OR Homosex* OR GLB* OR nonbinary OR Agender OR Aromantic OR Bigender OR Demigender OR Demisexual OR Multisexual OR “Third gender” OR Two*spirit OR (DE "Gender Nonconforming" OR DE "Gender Identity" OR DE "Cisgender" OR DE "Gender Nonbinary" OR DE "Gender Nonconforming" OR DE "LGBTQ" OR DE "Two-Spirit" OR DE "Gender Nonbinary" OR DE "Sexual Minority Groups") OR (DE "LGBTQ" OR DE "Asexuality" OR DE "Bisexuality" OR DE "Homosexuality" OR DE "Intersex" OR DE "Transgender")

AND

**Sexual Violence.** “Sexual violence” OR “sexual coercion” OR “sexual assault” OR Rape OR “Sexual Abuse” OR “Image W3 abuse” OR “Sexual Harassment” OR “Child* sexual abuse” OR “Sexual *victimization” OR “Incapacitated assault” OR “sexual* violen*” OR “date rape” OR “intimate partner violence” OR “partner violence” OR IPV OR “partner abus*” OR “dating violence” OR DE "Sexual Violence" OR DE "Sexual Harassment" OR DE "Sexual Coercion"

AND

**Help-seeking.** “Help seeking” OR “Seeking help” OR “Information seeking” OR “Assistance seeking” OR support OR “Mental health” OR “Service access" OR “Service use*” OR “Social support” OR reporting OR police OR hotline OR Disclos* OR Counselling OR Therapy OR “self-help” OR “support groups” OR “care seeking” OR DE "Help Seeking Behavior" OR DE "Health Care Seeking Behavior" OR DE "Self-Help Techniques"

**Web of Science**

**LGBTQ+.** LGBTQ+ OR LGB* OR “Non-heterosex*” OR “Non-monosex*” OR “Sexual minori*” OR SGM OR Bisexual* OR Pansexual* OR Queer* OR MSM OR “Men who have sex with men” OR WSW OR “Women who have sex with women” OR “Diverse Sex*” OR “Same*Sex” OR “Sexual and gender minorit*” OR Gay* OR Lesbian* OR Asexual* OR “Gender fluid*” OR Genderqueer OR “Gender nonconform*” OR “Non-binar*” OR Trans* OR “Gender minorit*” OR “Gender varian*” OR Homosex* OR GLB* OR nonbinary OR Agender OR Aromantic OR Bigender OR Demigender OR Demisexual OR Multisexual OR “Third gender” Two*spirit

AND

**Sexual violence.** “Sexual violence” OR “sexual coercion” OR “sexual assault” OR Rape OR “Sexual Abuse” OR “Image W3 abuse” OR “Sexual Harassment” OR “Child* sexual abuse” OR “Sexual *victimization” OR “Incapacitated assault” OR “sexual* violen*” OR “date rape” OR “intimate partner violence” OR “partner violence” OR IPV OR “partner abus*” OR “dating violence”

AND

**Help-seeking.** “Help seeking” OR “Seeking help” OR “Information seeking” OR “Assistance seeking” OR support OR “Mental health” OR “Service access" OR “Service use*” OR “Social support” OR reporting OR police OR hotline OR Disclos* OR Counselling OR Therapy OR “self-help” OR “support groups” OR “care seeking”

**Appendix B: Hierarchy of reasons for study exclusion**

a. Incorrect population (e.g., non LGBTQ+ or not having experienced sexual violence, participants under 18 years old).
b. Study is not specific to sexual violence.
c. Does not address barriers and facilitators to help-seeking for sexual violence survivors.
d. Study is not primary research.

**Appendix C: Quality Appraisal**

Qualitative studies were appraised using the CASP checklist. The questions are listed as follows:

1. Was there a clear statement of the aims of the research?
2. Is a qualitative methodology appropriate?
3. Was the research design appropriate to address the aims of the research?
4. Was the recruitment strategy appropriate to the aims of the research?
5. Was the data collected in a way that addressed the research issue?
6. Has the relationship between researcher and participants been adequately considered?
7. Have ethical issues been taken into consideration?
8. Was the data analysis sufficiently rigorous?
9. Is there a clear statement of findings?
10. How valuable is the research

| Source | 1 | 2 | 3 | 4 | 5 | 6 | 7 | 8 | 9 | 10 |
| --- | --- | --- | --- | --- | --- | --- | --- | --- | --- | --- |
| Mennicke et al. (2023). | Y | Y | Y | Y | Y | Y | C | Y | N | Y |
| Walsh et al. (2024) | Y | Y | Y | Y | Y | C | Y | Y | Y | Y |
| Tan et al. (2021) | Y | Y | Y | Y | Y | Y | Y | Y | Y | Y |
| Guadalupe-Diaz & Jasinski (2017) | Y | Y | Y | Y | Y | Y | Y | Y | Y | Y |
| Carlisle & Schmitz (2023) | Y | Y | Y | Y | Y | Y | Y | Y | Y | Y |
| Hereth (2021) | Y | Y | Y | Y | Y | C | C | Y | Y | Y |
| Strauss Swanson & Szymanski (2020) | Y | Y | Y | N | N | Y | C | Y | Y | Y |
| Fernández-Rouco et al. (2017) | Y | Y | Y | C | N | N | N | N | Y | Y |
| Meyer (2020) | Y | Y | Y | Y | Y | Y | C | Y | Y | Y |
| Sherman et al. (2022) | Y | Y | Y | Y | Y | Y | Y | Y | Y | Y |
| Brandt & Stults (2023) | Y | Y | Y | Y | Y | Y | Y | Y | Y | Y |
| Asseervatham et al. (2023) | Y | Y | Y | Y | Y | Y | Y | Y | Y | Y |
| Anderson & Overby. (2020) | Y | Y | Y | Y | Y | Y | Y | Y | Y | Y |
| Neves et al. (2023) | Y | Y | Y | N | Y | C | Y | Y | Y | Y |
| Turner & Hammersjö. (2024) | Y | Y | Y | Y | Y | C | Y | Y | Y | Y |
| Mgolozeli & Duma (2020) | Y | Y | Y | Y | Y | Y | Y | Y | Y | Y |
| de Heer et al. (2023) | Y | Y | Y | Y | Y | Y | C | Y | Y | Y |
| Bedera et al. (2023) | Y | Y | Y | Y | Y | Y | C | Y | Y | Y |
| Coston. (2019). | Y | Y | Y | Y | Y | C | C | Y | Y | Y |
| Hawkey et al. (2021) | Y | Y | Y | Y | Y | Y | Y | Y | Y | Y |
| Oueis et al. (2024) | Y | Y | Y | N | Y | C | Y | Y | Y | Y |
| Matsuzaka & Koch. (2019) | Y | Y | Y | N | Y | Y | Y | Y | Y | Y |
| Jackson et al. (2017) | Y | Y | Y | Y | Y | N | Y | Y | Y | Y |
| Scorgie et al. (2013) | Y | Y | Y | Y | Y | C | Y | Y | Y | Y |
| Witcomb & Cooper (2023) | Y | Y | Y | C | Y | Y | Y | Y | Y | Y |
| Wang (2011). | Y | Y | Y | Y | Y | Y | C | C | Y | Y |
| Donne et al. (2018) | Y | Y | Y | Y | Y | C | Y | Y | Y | Y |
| Harris & Dunn (2019). | Y | Y | Y | Y | Y | Y | Y | Y | Y | Y |
| Braun et al. (2009) | Y | Y | Y | Y | C | C | C | Y | Y | Y |
| LaRosa et al. (2024) | Y | Y | Y | C | Y | Y | Y | Y | Y | Y |

Mixed-methods studies were appraised using the MMAT. Questions are as follows:

1. Are there clear research questions
2. Is there an adequate rationale for using a mixed methods design to address the research question?
3. Is the qualitative approach appropriate to answer the research question?
4. Are the qualitative data collection methods adequate to address the research question?
5. Are the findings adequately derived from the data?
6. Is the interpretation of results sufficiently substantiated by data?
7. Is there coherence between qualitative data sources, collection, analysis and interpretation?
8. Is the sampling strategy relevant to address the research question?
9. Is the sample representative of the target population?
10. Are the measurements appropriate?
11. Is the risk of nonresponse bias low?
12. Is the statistical analysis appropriate to answer the research question?
13. Is there an adequate rationale for using a mixed methods design to address the research question
14. Are the different components of the study effectively integrated to answer the research question?
15. Are the outputs of the integration of qualitative and quantitative components adequately interpreted?
16. Are divergences and inconsistencies between quantitative and qualitative results adequately addressed?
17. Do the different components of the study adhere to the quality criteria of each tradition of the methods involved?

| Source | 1 | 2 | 3 | 4 | 5 | 6 | 7 | 8 | 9 | 10 | 11 | 12 | 13 | 14 | 15 | 16 | 17 |
| --- | --- | --- | --- | --- | --- | --- | --- | --- | --- | --- | --- | --- | --- | --- | --- | --- | --- |
| Salter et al. (2021). | Y | Y | Y | Y | Y | Y | Y | C | C | Y | Y | Y | Y | Y | Y | Y | Y |
| Hequembourg et al. (2021) | Y | Y | Y | Y | Y | Y | Y | Y | Y | Y | Y | Y | Y | Y | N | Y | Y |
| Flanders et al. (2020) | Y | Y | Y | Y | Y | Y | Y | Y | Y | Y | Y | Y | Y | Y | Y | Y | Y |
| Bowling et al. (2024) | Y | Y | Y | Y | Y | Y | Y | Y | Y | Y | Y | Y | Y | Y | Y | Y | Y |

Quantitative studies were assessed using the Quality Review and Critical Appraisal’ method, which involves scoring based questions as follows;

1. Clearly stated aims
2. Participant eligibility and recruitment strategy clearly documented
3. Main features of population/design described
4. Non-responders (and non-participants) described
5. Presence of a control group
6. Main limitations identified and acceptable
7. Sample size justified
8. No evidence of selective reporting of results
9. Statistical methods described
10. Statistical methods appropriate
11. Measures relevant, validated and described adequately
12. Results discussed adequately

In cases where the answer is yes, a score of two is assigned, a partial yes is scored 1 while no, or don’t know, is scored a 0. Total scores, as per the last column, indicate quality as all included quantitative studies scored above 17, they can be considered good quality.

| Source | 1 | 2 | 3 | 4 | 5 | 6 | 7 | 8 | 9 | 10 | 11 | 12 | T |
| --- | --- | --- | --- | --- | --- | --- | --- | --- | --- | --- | --- | --- | --- |
| Grocott et al. (2023) | 2 | 2 | 2 | 1 | 0 | 1 | 2 | 2 | 2 | 2 | 2 | 2 | 20 |
